# CD4/CD8 Ratio and KT Ratio Predict Yellow Fever Vaccine Immunogenicity in HIV-Infected Patients

**DOI:** 10.1371/journal.pntd.0005219

**Published:** 2016-12-12

**Authors:** Vivian I. Avelino-Silva, Karina T. Miyaji, Peter W. Hunt, Yong Huang, Marisol Simoes, Sheila B. Lima, Marcos S. Freire, Helio H. Caiaffa-Filho, Marisa A. Hong, Dayane Alves Costa, Juliana Zanatta C. Dias, Natalia B. Cerqueira, Anna Shoko Nishiya, Ester Cerdeira Sabino, Ana M. Sartori, Esper G. Kallas

**Affiliations:** 1 Department of Infectious and Parasitic Diseases, School of Medicine, University of Sao Paulo, Sao Paulo, Brazil; 2 Department of Medicine, University of California San Francisco, San Francisco, CA, United States of America; 3 Department of Bioengineering and Therapeutic Sciences, School of Pharmacy, University of California, San Francisco, San Francisco, California, United States of America; 4 Fundação Oswaldo Cruz, Rio de Janeiro, Brazil; 5 Instituto Adolfo Lutz, São Paulo, Brazil; 6 Laboratory of Medical Investigation LIM-3, School of Medicine, University of Sao Paulo, Sao Paulo, Brazil; 7 Division of Clinical Immunology and Allergy, School of Medicine, University of Sao Paulo, Sao Paulo, Brazil; 8 Fundação Pro-Sangue Hemocentro de Sao Paulo, Sao Paulo, Brazil; Naval Medical Research Center, UNITED STATES

## Abstract

**Background:**

HIV-infected individuals have deficient responses to Yellow Fever vaccine (YFV) and may be at higher risk for adverse events (AE). Chronic immune activation–characterized by low CD4/CD8 ratio or high indoleamine 2,3-dioxygenase-1 (IDO) activity—may influence vaccine response in this population.

**Methods:**

We prospectively assessed AE, viremia by the YFV virus and YF-specific neutralizing antibodies (NAb) in HIV-infected (CD4>350) and -uninfected adults through 1 year after vaccination. The effect of HIV status on initial antibody response to YFV was measured during the first 3 months following vaccination, while the effect on persistence of antibody response was measured one year following vaccination. We explored CD4/CD8 ratio, IDO activity (plasma kynurenine/tryptophan [KT] ratio) and viremia by Human Pegivirus as potential predictors of NAb response to YFV among HIV-infected participants with linear mixed models.

**Results:**

12 HIV-infected and 45-uninfected participants were included in the final analysis. HIV was not significantly associated with AE, YFV viremia or NAb titers through the first 3 months following vaccination. However, HIV–infected participants had 0.32 times the NAb titers observed for HIV-uninfected participants at 1 year following YFV (95% CI 0.13 to 0.83, p = 0.021), independent of sex, age and prior vaccination. In HIV-infected participants, each 10% increase in CD4/CD8 ratio predicted a mean 21% higher post-baseline YFV Nab titer (p = 0.024). Similarly, each 10% increase in KT ratio predicted a mean 21% lower post-baseline YFV Nab titer (p = 0.009). Viremia by Human Pegivirus was not significantly associated with NAb titers.

**Conclusions:**

HIV infection appears to decrease the durability of NAb responses to YFV, an effect that may be predicted by lower CD4/CD8 ratio or higher KT ratio.

## Author Summary

Yellow Fever (YF) vaccine is considered one of the most effective vaccines ever produced. However, previous studies suggest that HIV impairs YF vaccine response. In this study, we assessed if HIV infection impacts the risk of adverse events and could reduce antibody response to YF vaccine. We explored if laboratory markers of persistent inflammation, frequently present among HIV-infected patients, could predict antibody response to YF vaccine in this population. We found that HIV had no significant effect on adverse events or levels of antibodies through 3 months after vaccination, but this may be limited by the small sample size of 12 HIV-infected and 45-uninfected participants in the study. However, we were able to show that, compared to HIV-uninfected participants, HIV–infected patients had lower antibody titers 1 year following YF vaccine even after statistical adjustment for the potential effects of sex, age and prior vaccination. Persistent inflammation seems to reduce YF vaccine antibody response in HIV-infected participants. In conclusion, HIV-infected individuals have impaired antibody response to YFV due to a poorer persistence of antibodies, despite a seemingly normal initial response. HIV-infected patients at permanent or recurring risk of YF infection may benefit from a booster dose of YF vaccine.

## Introduction

Effective antiretroviral treatment (ART) drastically improved clinical outcomes for people living with HIV. However, these patients still present increased risk of death, higher prevalence of comorbidities, and impaired responses to vaccines [1–6]. Prior studies have shown impaired Yellow Fever vaccine (YFV) immunogenicity among HIV-infected persons is associated with detectable HIV viral load (VL) [7–12] and lower CD4 T cell counts [11]. However, it is still unclear whether reduced YFV antibody response among HIV-infected individuals is caused by a blunted initial response, decreased persistence of antibodies, or both. Moreover, predictors of YFV immunogenicity among patients with effective and early ART are not well known.

More recently, studies including patients with early initiation of ART have suggested a negative effect of persistent immune activation on responses to Influenza vaccine [13, 14], *Neisseria meningitis* vaccine [15] and YFV [16, 17] in both HIV-infected and–uninfected individuals. This is consistent with previous studies that demonstrate excessive immune activation and inflammation predict residual morbidity and mortality in treated HIV-infected patients [18–20]. A range of biomarkers has been used in different settings to quantify persistent immune activation [20]. One increasingly appraised indirect biomarker is the ratio of CD4 to CD8 T lymphocytes, or CD4/CD8 ratio. Previous studies have shown that CD4/CD8 ratio correlates with markers of CD8 T cell activation, and a lower CD4/CD8 ratio predicts higher risk of non-Aids events and mortality among ART-treated HIV-infected patients [21–23]. Furthermore, a low CD4/CD8 ratio is also strongly associated with the activity of Indoleamine 2,3-dioxygenase-1 (IDO), an enzyme expressed by activated myeloid cells in HIV and other inflammatory conditions that causes adaptive immune defects. IDO catabolizes tryptophan (T) to kynurenine (K) and other metabolites that may contribute to proliferative lymphocyte defects, regulatory T cell expansion, microbial translocation and immune activation in treated HIV infection [24]. Therefore, elevated IDO activity (as measured by plasma KT ratio) may also indicate adaptive immune dysfunction in this population. Finally, chronic co-infection with Human Pegivirus has been associated with reduced innate and adaptive immune activation among HIV-infected patients in prior studies [25–27].

An additional relevance of YFV in HIV-infected patients concerns the theoretic higher risk of YFV-associated severe adverse events (AE) in this population [28, 29]. YFV is produced from the 17D or 17DD attenuated viral strains, and although mechanisms for YFV-associated AE are not completely elucidated, immunosuppressed patients and persons at extremes of age are considered at increased risk [28, 30]. It is hypothesized that immune response fails to contain the vaccine virus replication, typically seen only in the first 3–5 days after vaccination, leading to uncontrolled viral dissemination and clinical disease [31].

In this study, HIV-infected patients and controls referred to receive YFV were enrolled and prospectively followed for 1 year after vaccination. We addressed clinical and laboratory AE and viremia by the YFV virus. In addition, we investigated if HIV status was associated with titers of Yellow Fever (YF)-specific neutralizing antibodies (NAb) in the first 3 months following vaccination (henceforth defined initial YFV immunogenicity) and one year following vaccination (henceforth defined persistence of YFV immunogenicity). Finally, we explored if correlates of immune activation including CD4/CD8 ratio, KT ratio, and Human Pegivirus co-infection predict YFV response among HIV-infected subjects.

## Methods

### Study population

Subjects aged 18 years old and older who were referred to receive YFV at Clinics Hospital in Sao Paulo, Brazil, between October 2011 and April 2014 were screened for participation.

Potential participants were evaluated by an attending physician who determined whether YFV was indicated based on risk of exposure to wild YF and YFV contraindications as defined by National Guidelines. The Guidelines do not recommend YFV to pregnant and breastfeeding women, subjects under immunosuppressive medications and patients with conditions such as cancer and thymus dysfunction. HIV-infected patients with a CD4 T cell count above 350/ml measured in the previous 4 months were considered eligible for vaccination. At enrollment, HIV-negative persons underwent a rapid HIV-test.

For both groups, participants with immunosuppressive conditions other than HIV infection were excluded. These included diabetes, chronic liver or kidney diseases, any type of cancer (except resolved Kaposi Sarcoma), and use of systemic immunosuppressive therapy in the last 3 months. Female participants in reproductive age underwent a pregnancy test at enrollment.

### Study procedures

**Fig 1:**
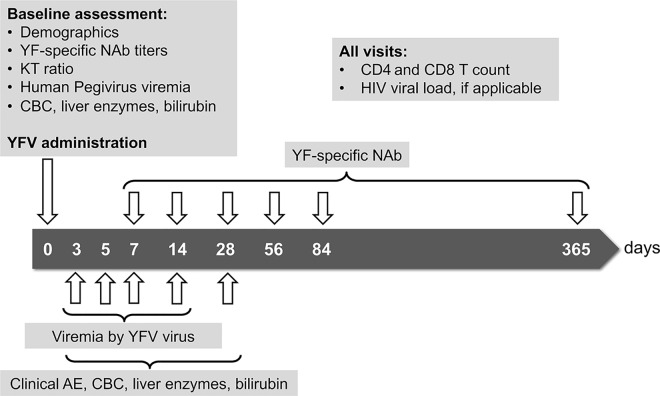
Overview of study procedures. YFV, Yellow Fever vaccine; NAb, neutralizing antibodies; KT, kynurenine/tryptophan; CBC, complete blood count; HIV, human immunodeficiency virus; AE, adverse events

At enrollment, medical history and date of previous YFV was obtained, if applicable. A blood sample was collected for assessment of baseline complete blood count and liver enzymes, CD4 and CD8 T cell counts, plasma KT ratio and Human Pegivirus viremia. HIV-infected participants had HIV VL measured at baseline and all subsequent visits. Participants were followed on days 3, 5, 7, 14, 28, 56, 84, and 365 after vaccination. We measured viremia by the YFV virus on days 3, 5, 7, and 14 after vaccination, and measured titers of YF-specific NAb at baseline and on days 7, 14, 28, 56, 84 and 365 after vaccination. We also collected data on spontaneous and solicited clinical AE, as well as laboratory AE on visits 3, 5, 7, 14 and 28 after vaccination. We measured CD4, CD8 T cells and CD4/CD8 ratio in all visits (Fig 1).

Clinical and laboratory AE were assessed as binary variables, defined as positive if the participant had any clinical AE, or any clinically significant laboratory AE at visits 3, 5, 7, 14 or 28, and negative otherwise. Laboratory AE were considered clinically significant if graded ≥2 according to the National Institutes of Allergy and Infectious Diseases’ Division of AIDS Table for Grading the Severity of Adult and Pediatric AE [32]. Viremia by YFV virus was assessed both as numeric and binary variable. The binary variable for YFV viremia was defined as positive if the participant had a detectable measurement (>200 copies/ml) on days 3, 5, 7 or 14 after vaccination, and negative otherwise.

### Laboratory methods

The HIV VL was determined by reverse-transcriptase (RT)-PCR using Amplicor HIV-1 Monitor Test (Roche Diagnostic Systems, NJ, USA), with a lower detection limit of 40/mm^3^. CD4 and CD8 T cell counts were determined by flow cytometry (FACSCalibur, BD Biosciences, CA, USA) using Multitest reagent (BD Biosciences).

Kynurenine and tryptophan were quantified on cryopreserved plasma samples by liquid chromatography–tandem mass spectrometry as previously described [33].

Human Pegivirus RNA was extracted from 140μl serum samples using QIAamp Viral RNA Mini Kit (QIAGEN Inc., California, USA), according to manufacturer’s instructions. A 5μl aliquot of extracted RNA was used to perform qRT-PCR with SuperScript III Platinum One-Step Quantitative RT-PCR System with ROX kit (Life Technologies), with primers and a TaqMan probe that amplified and quantified a fragment of 72-bp of the 5' untranslated region (5'UTR). The reaction was made with 0.5μl of SuperScript III RT/Platin Taq Mix, 12.5μl of 2X Reaction Mix with ROX, 0.75μl of 10μM Forward primer RTG1 (5’GTGGTGGATGGGTGATGACA3’) (Sigma), 1.25μl of 10μM Reverse primer RTG2 (5’GACCCACCTATAGTGGCTACCA3’) (Sigma), 0.4μl of 25 μM TaqMan probe ([6’FAM]CCGGGATTTACGACCTACC [TAMRA-6-FAM]) (Life Technologies), and reaction final volume of 25μl was completed with DEPC-treated water. cDNA synthesis was performed during the first 15 minutes at 50°C. After 2 minutes at 95°C, amplification and quantification were performed during 40 cycles with the following times and temperatures: 95°C, 15 seconds; 60°C, 30 seconds. The reading of FAM fluorescence was made during annealing period at 60°C.

For measurement of YFV viremia, total RNA was extracted from 140μl of plasma using QIAamp RNA Blood Mini Kit (Qiagen, Hilden, Germany) and eluted in 60μl of elution buffer. cDNA was obtained through RT reaction using 10μl of extracted RNA, 300ng of random primer (Amersham Biosciences, Piscataway, NJ, USA); 10U/μl of Super Script II RT (Invitrogen, Carlsbad, CA, USA) in a buffer solution with 0.25U/μl of ribonuclease inhibitor (Invitrogen) and 0.5mM deoxyribonucleotide triphosphates (Invitrogen), at final volume of 20μl. The reaction was incubated at 45°C for 90 minutes. Five μl of cDNA was added to 20μl of TaqMan Master Mix (Applied Biosystems, Foster City, CA, USA) and amplified by RT-PCR using the following primers and probe: (YF-NS5_F) 5’-GCACGG ATGTAACAGACTGAAGA-3’; (YF-NS5_R) 5’-CCAGGCCGAACCTGTC AT-3’ and (YF-NS5Probe) 5’-FAM-CGACTGTGTGGTCCGGCCCATC-3’–TAMRA [34]. The product was amplified using optical detection system layout of BioRad ICycler for 45 cycles at the following settings: 10 min, 95°C; 45 cycles of 15s for 94°C and 60s for 60°C.

NAb titers against YF virus were measured by Plaque Reduction Neutralization Test (PRNT) performed at Virologic Technology Laboratory of Bio-Manguinhos (LATEV, FIOCRUZ, Rio de Janeiro) as previously described [16].

### Ethical aspects

The study was approved by the Ethics Committee at Clinics Hospital in University of Sao Paulo. Upon participation, all participants signed an informed consent form. HIV tests were performed with pre and post-test counseling, and all individual identifiable information was maintained in secured cabinets and electronic files.

### Statistical analysis

Groups were compared using Wilcoxon rank-sum test for continuous variables and Fisher’s exact test for categorical variables. Titers of YF-specific NAb, CD4 and CD8 T cell counts were log-transformed to approximate normal distribution, and antilog transformation was required for model interpretation.

The effect of HIV status on levels of YFV viremia, and on initial YFV immunogenicity were investigated using mixed models with robust standard errors, adjusted for age, sex, previous YFV and interaction between HIV and visits. The effect of HIV status on persistence of YFV immunogenicity was investigated using a linear regression model adjusted for age, sex, previous YFV and baseline values of YF-specific NAb titers.

The effects of T CD4 and T CD8 cell count, detectable HIV VL, CD4/CD8 ratio, KT ratio, and Human Pegivirus viremia on YF-specific NAb titers among HIV-infected patients were investigated using mixed models adjusted for age, baseline NAb titers and HIV VL. Correlations between NAb titers and CD4/CD8 ratio or KT ratio were explored using Spearman rank correlation.

For all analysis, we assumed a two-sided alpha error of 0.05. All analyses were performed in Stata version 13.1 (StataCorp. College Station, TX: StataCorp LP).

We calculated sample size based on the impact of HIV status on titers of YF-specific NAb in the first 3 months following vaccination, using estimates of effect size and standard deviation from a prior study published by our group [16]. Since the analysis plan included mixed models for repeated outcomes, we assumed a 20% reduction in error variance and estimated a final sample of 33 participants per group using conventional means comparison.

## Results

Between October 2011 and April 2014, 63 participants were enrolled. Enrollment of a greater number of participants was compromised due to high refusal rates, mainly because most potential participants were referred to receive the YFV for a scheduled trip to an YF endemic area, and were therefore planning to be out of town and unable to attend the study visits. In addition, many potential participants refused participation due to the busy visit schedule, particularly in the first 2 weeks of follow-up. After exclusion of five controls and one HIV-infected participant due to missing visits, our final cohort included 12 HIV-infected and 45 HIV-uninfected individuals. All participants in the HIV-infected group were men, 11 (92%) were under ART and 8 (67%) had undetectable (<40 copies/ml) HIV VL. Participants under ART with detectable HIV VL had a range of 43–1,982 HIV copies/ml, and the only untreated participant had 110,531 HIV copies/ml at baseline. HIV-infected participants were more likely than -uninfected participants to be male and tended to be younger; 4 (33%) reported a prior YFV at a median of 14.5 years before enrollment (interquartile range [IQR] 10.5–21.5); none had received more than one YFV shot. Among HIV-uninfected participants, 16 (36%) reported a prior YFV at a median of 12 years before enrollment (interquartile range [IQR] 11–20); 10 had received a single shot and 6 (13%) had received two YFV shots in lifetime. Groups had similar YF NAb titers at enrollment, whether all participants or only participants with a prior YFV were considered (Table 1). Baseline CD4 T cell count was high among HIV-infected participants (median 722 cells/mm^3^, [IQR] 526–795), although still significantly lower than controls (median 941 cells/mm^3^, IQR 807–1470; p = 0.003). As expected, CD4/CD8 ratio was lower among HIV-infected participants (median 0.7, IQR 0.5–0.8) compared to controls (median 1.6, IQR 1.3–2.6; p<0.0001). KT ratio was also higher in HIV-infected group, but the difference did not reach statistical significance (median 35.9 versus 31.3 nM/μM, p = 0.06). Viremia by Human Pegivirus was more prevalent in HIV-infected (67%) than in -uninfected group (27%, p = 0.016, Table 1).

**Table 1:** Baseline characteristics of participants*

	HIV-infected	Controls	p-value
	N = 12	N = 45	
Age–years	33 (30–47)	43 (31–62)	0.12
Male gender—N (%)	12 (100)	22 (49)	0.001
Previous YFV–N (%)	4 (33)	16 (36)	0.56
Single lifetime YFV dose	4 (33)	10 (22)	
Two lifetime YFV doses	0	6 (13)	
YF NAb, Log_10_mIU/mL			
All participants	0 (0–3.0)	2.4 (0–3.3)	0.48
Participants with prior YFV	3.3 (2.9–4.0)	3.4 (3.2–4.0)	0.71
CD4 T count–cells/mm^3^	722 (526–795)	941 (807–1470)	0.003
CD4/CD8 ratio	0.7 (0.5–0.8)	1.6 (1.3–2.6)	<0.0001
KT ratio–nM/μM	35.9 (33.4–43.9)	31.3 (27.5–37.7)	0.06
Viremia by Human Pegivirus–N (%)	8 (66.7)	12 (26.7)	0.016
Undetectable^#^ HIV VL–N (%)	8 (67)	-	-

HIV, human immunodeficiency virus; YFV, Yellow Fever Vaccine; YF NAb, Yellow Fever-specific neutralizing antibodies; KT, kynurenine/tryptophan; VL, viral load.

*Continuous variables are presented as medians and interquartile ranges.

^#^Considered undetectable if below 40 copies/ml.

**Fig 2:**
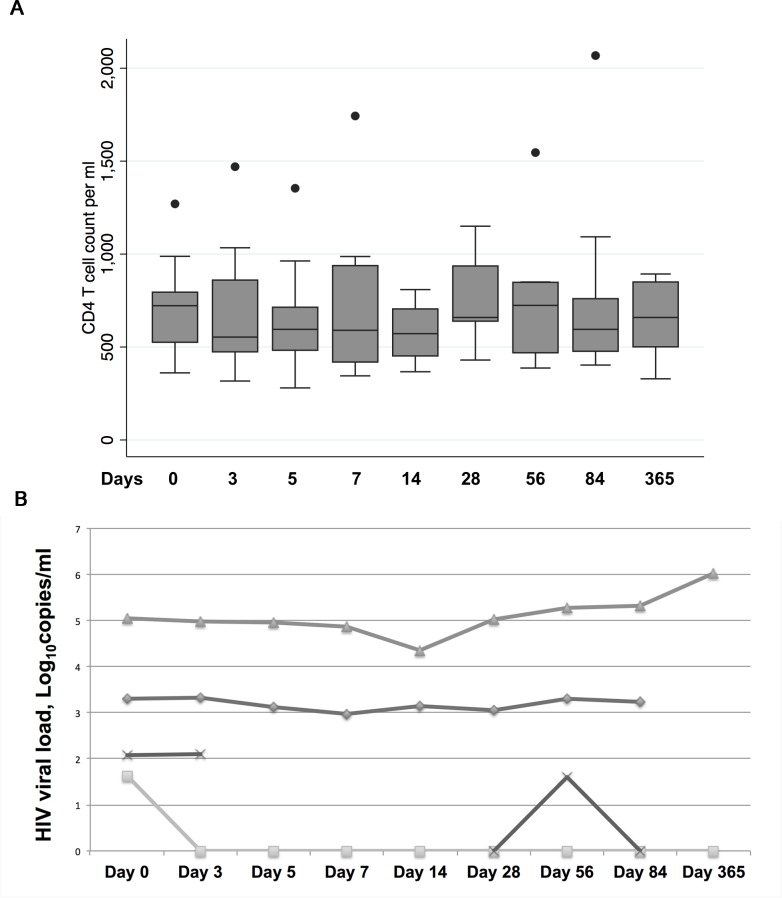
CD4+ T cell counts for HIV-infected participants (N = 12) across study visits (Panel A) and HIV VL across visits for participants with VL above the limit of detection at baseline (N = 4, Panel B). Individual subjects are represented by distinct lines in the graph.

During study visits, HIV-infected subjects had no substantial change in CD4 T cell count compared to baseline values (Fig 2A). HIV-infected participants who had detectable HIV VL at baseline had no meaningful change in HIV VL across visits (Fig 2B). Among the 8 participants with undetectable HIV VL at baseline, only one had a detectable value (51 copies/ml) at visit 84. HIV-infected participants had no change in ART status during follow-up.

### AE following vaccination and the effect of HIV on viremia by the YFV virus

Any clinical AE was reported by 6 (50%) participants in the HIV-infected group, and 22 (48.9%) controls. All reported clinical AE (local pain, tenderness and redness; nausea, myalgia, fatigue, dizziness and fever) were mild and self-limited, as were laboratory AE–anemia, neutropenia, lymphopenia, thrombocytopenia and liver enzymes elevation–which were detected in 3 (25%) individuals in the HIV-infected group, and 13 (28.9%) controls.

Viremia by the YFV virus was detected in at least one visit in 40% of HIV-infected participants and 34% of controls. Maximum detected viremia was 11210 copies/mL in one HIV-uninfected participant at day 5 after vaccination; in the HIV-infected group, highest measured viremia was also observed at day 5 (4197 copies/mL). HIV status was not statistically associated with levels of viremia by the YFV virus (p-value = 0.99 for the overall effect of HIV on YFV viremia on days 3, 5, 7 and 14).

### Effect of HIV status on initial YFV immunogenicity

At baseline, 4 HIV-infected participants (33%) and 17 controls (38%) had levels of NAb considered seropositive for a cutoff of 794 mUI/mL as defined by the referent laboratory [35]. If only participants with a previous YFV were considered, 3 HIV-infected (75%) and 15 controls (94%) had seropositive NAb titers. At 28 days after vaccination, all participants in both groups were seropositive for YF, and at one year following vaccination, 11 HIV-infected participants (92%) and 43 controls (96%) maintained seropositive YF-specific NAb.

We failed to find a statistically significant difference between groups defined by HIV status on initial YFV immunogenicity in either visit individually or overall in a mixed model adjusted for age, sex and previous YFV (Table 2). The model predicted lower YF-specific NAb titers for women; in average, women had 0.33 times the titers observed for men (95% CI 0.17–0.66, p = 0.002). As expected, compared to individuals without previous YFV, those who reported a previous YFV had higher NAb titers (fold change 13.69, 95% CI 7.12–26.30, p<0.001).

**Table 2:** Effect of HIV status on initial immunogenicity after YFV

	Predicted multiplicative effect in YF-specific NAb	95% CI	p-value
Age (per year increase)	1.00	0.98 to 1.02	0.82
Female sex	0.33	0.17 to 0.66	0.002
Previous YFV	13.69	7.12 to 26.30	<0.001
HIV status			
Overall HIV effect across visits			0.25
Baseline	0.19	0.03 to 1,24	0.08
Day 7	0.95	0.11 to 8.35	0.96
Day 14	0.60	0.10 to 3.55	0.57
Day 28	0.66	0.21 to 2.08	0.47
Day 56	0.67	0.19 to 2.35	0.53
Day 84	1.06	0.36 to 3.08	0.92

HIV, human immunodeficiency virus; YFV, Yellow Fever vaccine; NAb, neutralizing antibodies

Model adjusted for age, sex and previous YFV

### Effect of HIV status on persistence of YFV immunogenicity

Persistence of YFV immunogenicity was significantly lower in HIV-infected participants compared to controls in a mixed model adjusted for age, sex, previous YFV and baseline NAb titers. In average, HIV-infected individuals had 0.32 times the NAb titers observed for HIV-uninfected participants at one year after vaccination (95% CI 0.13–0.83, p = 0.021). We found no statistically significant effect of age, sex or previous YFV on persistence of NAb (Table 3).

**Table 3:** Effect of HIV status on persistence of YFV immunogenicity

	Predicted multiplicative effect in YF-specific NAb	95% CI	p-value
Age (per year increase)	1.01	0.96 to 1.07	0.57
Female sex	0.54	0.12 to 2.50	0.42
Previous YFV	1.27	0.25 to 6.48	0.77
Baseline YF-specific NAb	1.08	0.63 to 1.85	0.78
HIV infection	0.32	0.13 to 0.83	0.021

HIV, human immunodeficiency virus; YFV, Yellow Fever vaccine; NAb, neutralizing antibodies

Model adjusted for age, sex, previous YFV and baseline values of YF-specific NAb titers

### Predictors of YFV immunogenicity among HIV-infected participants

In the exploratory analysis restricted to HIV-infected patients, higher CD4/CD8 ratio and lower KT ratio predicted higher YF-specific NAb titers; in average, for each 10% increase in CD4/CD8 ratio, post-baseline NAb titers were 21% higher (95% CI 3–38%, p = 0.024), and for each 10% increase in KT ratio, post-baseline NAb titers were 21% lower (95% CI 5–37% lower, p = 0.009) after adjustment for age, baseline NAb titers and HIV VL. There was no evidence for an association between CD4 or CD8 T cell count and YFV immunogenicity (multiplicative effect per 10% increase 1.05, 95% CI 0.91–1.20, p = 0.469, and 0.95, 95% CI 0.86–1.04, p = 0.295, respectively) or between Human Pegivirus co-infection and YFV immunogenicity among HIV-infected individuals (fold change 0.65, 95% CI 0.09–4.47, p = 0.659, Table 4). Adjusted for age and baseline NAb titers, having detectable plasma HIV was associated with 60% lower YF-specific NAb titers (fold change 0.40, 95% CI 0.21–0.75, p = 0.004).

**Table 4:** Predictors of YFV immunogenicity in HIV-infected participants

	Unadjusted multiplicative effect in YF-NAb	95% CI	Adjusted multiplicative effect in YF-NAb^#^	95% CI	p-value
CD4 T count*	1.07	0.96 to 1.18	1.05^#^	-1.08 to 1.17	0.47
CD8 T count*	0.98	0.89 to 1.06	0.95^#^	0.86 to 1.04	0.30
CD4/CD8 ratio*	1.12	0.68 to 1.23	1.21^#^	1.03 to 1.38	0.024
Human Pegivirus	1.12	0.21 to 5.90	0.65^#^	0.09 to 4.47	0.66
KT ratio*	0.86	0.20 to 0.74	0.79^#^	0.63 to 0.95	0.009
Detectable HIV VL	0.36	0.20 to 0.67	0.40^§^	0.21 to 0.75	0.004

YFV, Yellow Fever vaccine; HIV, human immunodeficiency virus; NAb, neutralizing antibodies; KT, kynurenine/tryptophan; VL, viral load

*Effect per each 10% increase in the predictor

^#^Model adjusted for age, baseline NAb titers and HIV VL

^§^Model adjusted for age and baseline NAb titers

As to confirm the effects of CD4/CD8 ratio and KT ratio on NAb titers, we performed simple non-parametric correlation tests; as expected, CD4/CD8 ratio correlated positively with NAb titers in all time-points, with statistically significant correlation in visit 28 (Rho = 0.74, p = 0.0139) and visit 365 (Rho = 0.9, p = 0.0374). Similarly, KT ratio correlated negatively with NAb titers in all time-points, with statistically significant correlation in visit 56 (Rho = -0.76, p = 0.0171).

## Discussion

In this prospective cohort of individuals receiving YFV, those with HIV had similar levels of YFV viremia and AEs as HIV-uninfected controls. Compared to controls, HIV-infected participants also had similar initial immunogenicity to YFV, measured by YF-specific NAb titers at 7, 14, 28, 56, and 84 days after vaccination, adjusted for age, sex and previous YFV. However, HIV status was independently associated with lower persistence of YF-specific NAb titers one year after vaccination. In the analysis of predictors of immunogenicity among HIV-infected participants, lower CD4/CD8 ratio, higher KT ratio and detectable HIV VL were associated with lower YF-specific NAb titers. There was no evidence for an association between viremia by Human Pegivirus, CD4 and CD8 T cell counts and YF-specific NAb titers among HIV-infected individuals.

Earlier studies of YFV immunogenicity including HIV-infected patients in the pre-ART period or in the initial phases of ART had demonstrated that CD4 T cell count and HIV VL predicted YF-specific NAb titers [7–12]. However, in the current era of early ART initiation, more HIV-infected patients are expected to have undetectable HIV VL and higher CD4 T cell counts. In our study, despite the elevated CD4 T cell count and high proportion of ART-suppressed individuals, HIV status was still associated with lower persistence of YF-specific NAb titers following an apparently adequate initial immunogenicity. Our results are consistent with prior publications suggesting that HIV-infected subjects still present lower responses to vaccines [1, 6, 16]. In addition, while the START and TEMPRANO trials demonstrated that earlier ART initiation dramatically reduces the risk of infectious outcomes, there was still a substantial risk of infectious outcomes in the immediate ART arms [36, 37]. Thus there are likely to be subtle immune defects that persist despite early ART initiation. Our study provides potentially important insights into mechanisms that might contribute to this persistent risk of infectious complications as well as point of care diagnostics that might identify patients at highest risk. For example, higher plasma KT ratio–a marker of IDO activity–strongly predicted lower YFV Nab titers after vaccination. While our observational study cannot assess causality, the fact that IDO-generated tryptophan catabolites suppress lymphocyte proliferation and function provides a plausible mechanistic pathway of its detrimental effect on vaccine responsiveness and, more broadly, adaptive immunity. Indeed, higher IDO activity has already been shown to predict increased mortality in several cohorts of ART-suppressed HIV-infected individuals [38–40]. While higher IDO activity might simply be a surrogate of other immunologic defect causally associated with impaired B cell function (e.g., the extent of T follicular helper cell infection and/or dysfunction in lymphoid tissues), a potential causal role for IDO activity in impairing vaccine responsiveness is plausible. Interestingly, high KT ratio was one of the strongest immunologic correlates of low CD4/CD8 ratio in another recent study of ART-suppressed individuals [21], suggesting that this biomarker–already obtained as part of routine clinical care—might help identify individuals with highest risk of impaired vaccine responses and adaptive immune defects.

Collectively, our findings further encourage the development of therapeutic interventions to reduce immune activation in ART-treated HIV-infected individuals [18, 20]. Early ART initiation is a well-recognized, yet insufficient strategy to normalize persistent immune activation [41], and additional strategies including inhibition of IDO activity are currently under study [42].

While effective interventions to inhibit immune activation are not available for clinical use, another important implication of our findings is the potential to substantiate recommendations for a booster dose of YFV for HIV-infected individuals at permanent or recurring risk of wild YF. In a recent publication, the Advisory Committee on Immunization Practices from Centers for Disease Control and Prevention published recommendations for YFV, suggesting HIV-infected individuals may benefit from a booster vaccination, which would not be recommended in routine circumstances due to the high immunogenicity and durability of YFV in the general population [43]. Our results suggest that a booster YFV may be beneficial even for HIV-infected individuals with high CD4 T cell counts. Since lower persistence of NAb was observed one year after YF vaccination, and AE following a booster dose of YFV are rare [28, 29], either periodic monitoring of YF-NAb or administration of a booster YFV dose could be recommended for HIV-infected individuals at permanent or recurring risk of wild YF as early as one year after primary vaccination. Additional studies are necessary to determine the durability of immunogenicity after a booster vaccination in this population.

Because all included participants received YFV as indicated due to potential risk of exposure to wild-type virus, we cannot rule out that natural exposure, rather than YFV alone, partially accounted for the observed NAb titers. However, most participants were residents in non-endemic areas and received YFV due to temporary visits to endemic regions with low risk of natural exposure. Furthermore, this potential competing immune stimulus would likely occur non-differentially regarding HIV status. Consequently, we do not believe our results are substantially compromised by natural exposure to wild YF virus.

Due to the small sample size, our results must be interpreted with caution. The model addressing predictors of YFV immunogenicity among HIV-infected participants included only 12 individuals followed longitudinally with 7 repeated outcome measurements. Although statistical methods for longitudinal analysis typically reduce error variance and improve power, this exploratory analysis needs confirmation in larger samples and different settings. In addition, our study was likely underpowered to provide definitive conclusions regarding the effect of HIV status on initial YFV response, on risk of AE (in particular rare severe AE) and on viremia by the YFV virus. Sensitivity for detection of AE was enhanced in our study by the measurement of solicited clinical AE, laboratory assessment of potential hematological or biochemical abnormalities, and systematic measurement of YFV viremia. Therefore, although not definitive, our findings provide important information on YFV clinical and laboratory adverse events, as well as vaccine virus kinetics among HIV-infected participants.

Our study may also be underpowered to detect significant effects of viremia by Human Pegivirus, CD4 and CD8 T cell counts on YF-specific NAb titers among HIV-infected participants. Our study included HIV-infected individuals with a very high range of CD4 T cell count, and we cannot rule out that CD4 T cell count would still predict YFV immunogenicity among patients with wider CD4 T cell count variability. Finally, we used a single measurement of RT-PCR to determine Human Pegivirus co-infection, and were unable to discriminate recent unresolved infections from chronic infections.

In conclusion, HIV-infected individuals have impaired NAb response to YFV due to a poorer persistence of antibodies, despite a seemingly normal initial response. Immune activation seems to reduce YFV immunogenicity, consistent with the observation that immune activation markers are useful predictors of clinical outcomes in the current era of HIV care [21, 22, 38]. A booster dose of YFV, although not recommended in routine circumstances, may be beneficial for HIV-infected individuals at permanent or recurring risk of wild YF.

## Supporting Information

S1 Checklist STROBE checklist of items included in reports of *cohort studies* (DOC)
